# Biochemical responses and oxidative stress in *Francisella tularensis *infection: a European brown hare model

**DOI:** 10.1186/1751-0147-53-2

**Published:** 2011-01-13

**Authors:** Hana Bandouchova, Miroslav Pohanka, Kristina Vlckova, Veronika Damkova, Lucie Peckova, Jana Sedlackova, Frantisek Treml, Frantisek Vitula, Jiri Pikula

**Affiliations:** 1 Department of Veterinary Ecology and Environmental Protection, Faculty of Veterinary Hygiene and Ecology, University of Veterinary and Pharmaceutical Sciences Brno, Palackeho 1/3, 612 42 Brno, Czech Republic; 2 Centre of Advanced Studies and Department of Toxicology, Faculty of Military Health Sciences, University of Defence/Trebesska 1575, 50001 Hradec Kralove, Czech Republic; 3 Department of Biological and Biochemical Sciences, Faculty of Chemical Technology, University of Pardubice, Studentska 95, 532 10 Pardubice, Czech Republic; 4 Department of Infectious Diseases and Epidemiology, Faculty of Veterinary Medicine, University of Veterinary and Pharmaceutical Sciences Brno, Palackeho 1/3, 612 42 Brno, Czech Republic

## Abstract

**Background:**

The aim of the present study was to investigate biochemical and oxidative stress responses to experimental *F. tularensis *infection in European brown hares, an important source of human tularemia infections.

**Methods:**

For these purposes we compared the development of an array of biochemical parameters measured in blood plasma using standard procedures of dry chemistry as well as electrochemical devices following a subcutaneous infection with a wild *Francisella tularensis *subsp. *holarctica *strain (a single dose of 2.6 × 10^9 ^CFU *pro toto*).

**Results:**

Subcutaneous inoculation of a single dose with 2.6 × 10^9 ^colony forming units of a wild *F. tularensis *strain *pro toto *resulted in the death of two out of five hares. Plasma chemistry profiles were examined on days 2 to 35 post-infection. When compared to controls, the total protein, urea, lactate dehydrogenase, aspartate aminotransferase and alanine aminotransferase were increased, while albumin, glucose and amylase were decreased. Both uric and ascorbic acids and glutathione dropped on day 2 and then increased significantly on days 6 to 12 and 6 to 14 post-inoculation, respectively. There was a two-fold increase in lipid peroxidation on days 4 to 8 post-inoculation.

**Conclusions:**

Contrary to all expectations, the present study demonstrates that the European brown hare shows relatively low susceptibility to tularemia. Therefore, the circumstances of tularemia in hares under natural conditions should be further studied.

## Background

Tularemia is considered a re-emerging zoonosis [1-3] that is endemic under favourable environmental conditions [4]. The highly infectious Gram-negative bacterium *Francisella tularensis *has been reported to cause infection in a wide range of hosts including humans [5,6]. Much attention has also been paid to the role of haematophagous arthropods as potential vectors of this zoonosis [7]. Among wild animals, lagomorphs such as the European brown hare (*Lepus europaeus*) seem to be the most important in terms of public health concern [8-11]. The distribution of natural foci of tularemia was found to be dependent on the population density of the European brown hare [10]. This species of game is a very good indicator of the presence and activity of the causative agent, *F. tularensis*, in natural foci, and has been used routinely for the surveillance of this zoonosis by the State Veterinary Administration in some areas of the Czech Republic. It is even possible to plot a prediction map of the geographic distribution of tularemia using data on European brown hares [12]. Concomitantly with tularemia in hares, the incidence of human tularemia is also increasing [7], frequently as a result of handling tularemic hares [5,11,13].

Tularemia is also of interest as a model for the pathogenesis of intracellular bacteria [14]. *F. tularensis *infection confers oxidative stress upon target cells, and many of the host-defence mechanisms appear to be intended to counteract this stress [15]. Cells are equipped with defence mechanisms that provide protection via enzymatic activities or through low molecular weight antioxidants (LMWAs) acting as chemical scavengers and neutralizing reactive molecular species [16]. Interestingly, *F. tularensis *is capable of utilizing glutathione present in the cytosol of infected host cells. Cleavage of this antioxidant provides the essential source of cysteine required for intracellular multiplication of *Francisella *[17]. It was reported recently that the biochemical responses of various hosts may vary. There were marked differences in lipid metabolism in the course of tularemia in BALB/c mice and common voles. Hypertriglyceridemia and hypercholesterolemia developed in mice, while physiologically higher levels of triglycerides and cholesterol showed a decreasing tendency in common voles (*Microtus arvalis*). On the other hand, the total plasma antioxidant capacity gradually dropped to 81.5% in mice, while it increased to 130% after the infection in common voles. Significant correlations between tissue bacterial burdens and several biochemical parameters were found [18].

Experimental models of tularemia employ laboratory mice, in particular [15,19-23], while European brown hares have only been used exceptionally [24-26] despite their importance as a source of human infections. It was therefore the aim of the present study to investigate biochemical and oxidative stress responses to experimental *F. tularensis *infection in European brown hares. For these purposes we evaluated the dynamics of biochemical parameters measured in blood plasma using both standard procedures of dry chemistry and electrochemical devices.

## Materials and methods

### Experimental micro-organism

A wild strain of *Francisella tularensis *isolated from a European brown hare specimen from South Moravia in 2004 was used for experimental infections in this study. The isolate was subtyped as *Francisella tularensis *subsp. *holarctica *via the proteomic procedure [27]. Experimental infections were performed using a suspension of *F. tularensis *cells harvested from a culture growing on blood agar supplemented with L-cysteine using sterile physiological saline solution. After thorough mixing we measured the absorbance of the suspension at 605 nm using a spectrophotometer (Unicam Helios Gamma&Delta, Spectronic Unicam, United Kingdom) in order to determine the number of bacterial cells per unit volume according to McFarland's standard [28]. The number obtained was only approximate and was used to estimate the dilution necessary to achieve the dose required. The exact infectious dose was then determined by plating ten-fold serial dilutions and counting colony-forming units (CFU) in the suspension administered to experimental animals. Colonies were counted after 72 h of incubation at 37 °C. Virulence of the *F. tularensis *strain was tested by inoculation of BALB/c mice.

### Experimental animals

One-year-old European brown hares (*Lepus europaeus*) were purchased from the Hare Breeders' Association of the Czech Republic and a total of ten males were used for the study. They were fed standard granules for rabbits (without supplementation of anticoccidials) and high quality hay, and were provided with drinking water *ad libitum*. At the start of the experiment the hares appeared healthy, were in an excellent nutritional state, and were certified free of tularemia and brucellosis based on agglutination tests.

### Experimental design

Experimental hares were allocated to the control and *F. tularensis*-inoculated groups (five specimens each) on a random basis. Biochemical responses, lipid peroxidation and levels of antioxidants were studied following subcutaneous infection of the inoculated group. Hares were inoculated into the dorsal trunk area with a single dose of 2.6 × 10^9 ^CFU *pro toto*. Blood for plasma chemistry profiles was collected every other day from days 0 to 16, and on days 24 and 35. The data from infected hares were then compared against values obtained from control hares from days 0 to 35 of the experiment plus healthy hares sampled prior to inoculation (n = 60). Blood was collected from the jugular vein using a heparinized set Omnican^® ^40 (Braun, Germany). Samples of blood were centrifuged immediately after collection, and the plasma was removed and frozen (-80 °C). Surviving hares were killed on day 35 post-inoculation. Necropsy was performed in hares that died or were euthanized in order to determine gross pathological findings and to collect organs aseptically (liver, spleen, lung, bone marrow and kidney). Tissue samples were also collected to 10% buffered formalin, treated using a routine histological technique and embedded in paraffin. Sections of 5μm were made of the paraffin blocks and stained with haematoxylin and eosin. Organ and blood samples were examined for the presence of *F. tularensis *by culture and the mouse inoculation test. Agglutination antibody titres were examined using a commercially available antigen (Bioveta a.s., Ivanovice na Hane, Czech Republic).

Experiments were performed in compliance with laws for the protection of animals against cruelty and were approved by the Ethical Committee of the University of Veterinary and Pharmaceutical Sciences Brno, Czech Republic.

### Assay of low molecular weight antioxidants by square wave voltammetry

Square wave voltammetry (SWV) was used to estimate low molecular weight antioxidants (LMWAs) in plasma samples as described previously [29]. The anodic current was measured in order to estimate the occurrence of compounds that are able to donate electrons, i.e., antioxidants [16]. The device EmStat (PalmSens, Houten, Netherlands) and screen-printed strips with platinum working (1 mm diameter, dot-shaped), silver/silver chloride reference and platinum auxiliary electrodes on a ceramic support (BVT, Brno, Czech Republic) were used throughout the experiments. The strips were washed with ethanol and water prior to use. EmStat was adjusted to the following parameters: applied potential in the range 0 - 1 V; potential step as well as potential amplitude 0.01 V; frequency 1 Hz. Measurement began by spreading 20 μl of plasma over the electrodes. Each strip was only used for one measurement in order to avoid hysteretic influences.

### Thiobarbituric acid reactive substances assay

Total thiobarbituric acid reactive species (TBARS) in plasma were assayed as described previously [30]. A stock solution of thiobarbituric acid (TBA) was prepared by diluting 67 mg of TBA in 1 ml dimethylsulphoxide and subsequently adding 9 ml of deionized water. One hundred μl of plasma were mixed with 200 μl ice cold 10% trichloroacetic acid and incubated in an ice bath for 15 minutes. The mixture was centrifuged at 3000 × g for 15 minutes in order to displace precipitated proteins. After centrifugation, 200 μl of supernatant were injected into a new tube and the same volume of TBA solution was added. Finally, the mixture was incubated in a boiling water bath for 10 minutes. A blank was prepared using the above-mentioned protocol with plasma replaced by physiological solution. After cooling to laboratory temperature, absorbance was measured against the blank at 532 nm.

### Biochemistry

Within a few days of collection, plasma was analysed using an automated analyser (SPOTCHEM™ EZ SP-4430, ARKRAY, Japan) for total proteins and albumin (g/l), creatinine (μmol/l), urea (mmol/l), uric acid (mmol/l), aspartate aminotransferase (μkat/l), alkaline phosphatase (μkat/l), alanine aminotransferase (μkat/l), lactate dehydrogenase (μkat/l), creatine kinase (μkat/l), total cholesterol (mmol/l), triglycerides (mmol/l), glucose (mmol/l) and total bilirubin (μmol/l).

### Statistical analysis

Statistical analyses were performed using Statistica for Windows 7.0 (StatSoft, Tulsa, OK, USA). Data normality and homogeneity of variances were evaluated by the Kolmogorov-Smirnov test and the Levene's test, respectively. One-way analysis of variance (ANOVA) and the nonparametric Kruskal-Wallis test were used for statistical comparisons. In the case of non-normal data distribution, nonparametric statistical analysis also included the Mann-Whitney *U *test. Values of *p *< 0.05 and *p *< 0.01 were considered statistically significant and highly significant, respectively, for all tests. Spearman rank order correlation analysis was employed to examine the relationship between low molecular weight antioxidants and plasma chemistry profiles.

## Results and discussion

There was no mortality in the control group during the study, while two of the five European brown hares from the *F. tularensis*-inoculated group died. Clinical signs of tularemia started to develop one day post-inoculation and included fever as high as 41 °C, lethargy and anorexia. Hares succumbed to the infection on days 5 and 9 post-inoculation. There was splenomegaly and microscopic examination of tissue slides revealed diffuse necroses in the spleen, focal necroses in the liver and moderate vacuolization of hepatocytes. Blood culture yielded positive results in samples collected from three, four and one hares on days 2, 4 and 6 post-inoculation, respectively. Bacteraemia was also confirmed using the above samples and the mouse inoculation test. Positive cultures were obtained from liver, spleen, lung, bone marrow and kidney tissues in the hare that died on day 5, while only spleen and bone marrow tissues were burdened by bacteria in the hare dying on day 9 post-inoculation. The remaining three hares were killed on day 35 post-inoculation. There were no gross and microscopic pathological findings in the surviving hares, and organs collected aseptically (liver, spleen, lung, bone marrow and kidney) were free of *F. tularensis *based on culture and the mouse inoculation test. Tube agglutination antibodies first occurred between days 8 to 10 and amounted up to the titre of 1:640 on day 35 post-inoculation.

Since we used only a single dose with approximately 2.6 × 10^9 ^colony forming units (CFU) *pro toto*, it was not the purpose of the present study to determine the LD_50 _of the *F. tularensis *infection in hares. However, the selected dose resulted in the death of two out of five inoculated hares and it seems that it was close to the LD_50 _for this mammalian species and the subcutaneous route of infection. The European brown hare may thus be considered a species of relatively low susceptibility to tularemia when exposed via this route because, for example, in the highly susceptible BALB/c mice and common voles the LD_50 _was calculated to be about 1 and 38 CFU, respectively [19]. Similar results of lower susceptibility to tularemia were obtained in a study when three European brown hares survived intramuscular or intraperitoneal inoculation of 1.0 × 10^9 ^bacteria of *F. tularensis *biovar *palaearctica *[24]. Authors of the study discussed these unexpected results by the possibility of lower virulence of the bacteria due to decapsulation of *F. tularensis *by solution of sodium chloride. Attenuation of the *F. tularensis *strain by *in vitro *passage could be another reason for the survival of experimental hares after an enormous infectious dose. It was, however, improbable because virulence of the experimental *F. tularensis *strain was tested by inoculation of BALB/c mice and provided results standard for this highly susceptible laboratory species [19]. Our results of low susceptibility of European brown hares to tularemia contrast with some other reports classifying hares as highly susceptible [26,31]. Experimental hares were infected by the subcutaneous route, which is clinically relevant because it imitates one of the natural routes of tularemia transmission via ticks that carry the agent. It was demonstrated that the numbers of *F. tularensis *cells fluctuate from 40 to 69 300 in infected ticks such as *Dermacentor reticulatus, D. marginatus *and *Ixodes ricinus *from natural foci of tularemia [7]. In light of this, however, fatal infection due to transmission of tularemia in this way would require a really heavy tick infestation.

**Figure 1:**
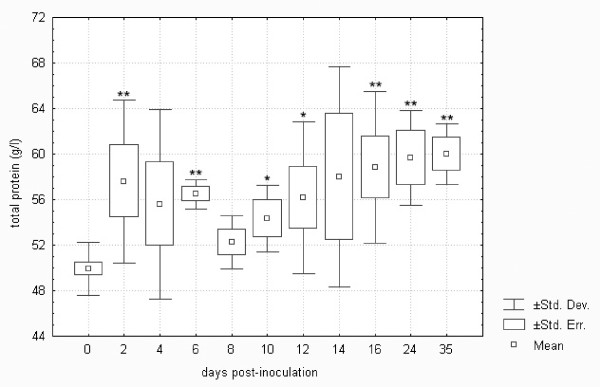
**Total plasma protein in European brown hares on individual days post-inoculation with *F. tularensis***. Group 0 represents the range of values obtained when measuring control hares throughout days 0 to 35 of the experiment plus healthy hares sampled prior to inoculation (n = 60); 2 to 35 represent groups of animals sampled on days 2 to 35 post-infection (n = 5 until day 4, n = 4 on days 6 and 8, and n = 3 from day 10 to 35); * = p < 0.05, ** = p < 0.01 when compared against control group 0.

**Figure 2:**
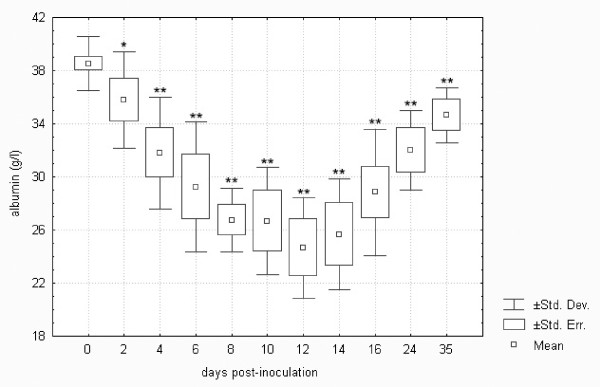
**Plasma albumin in European brown hares on individual days post-inoculation with *F. tularensis***. See Figure 1 for a detailed description of groups.

In terms of the development profile of the plasma biochemistry parameters in the control and *F. tularensis*-inoculated groups, no significant differences were found in total cholesterol, triglycerides and creatine kinase. Figures 1 and 2 demonstrate differences in the development profile of total protein and albumin. As shown, the levels of total protein were higher from day 2 to 35 post-infection by up to 120% when compared to the normal levels in healthy hares. On the other hand, albumin levels showed a decreasing trend, falling to as low as 60% of the normal levels from day 2 to 12 post-infection, and the reversal of the trend from day 14 to 35 was not sufficient enough to normalize the levels.

It is known that sepsis initiates a cascade of changes associated with substrate metabolism and a reprioritization of the normal catabolic and anabolic processes [32]. Increased levels of total protein are due to the production of the acute phase proteins such as α1-acid glycoprotein, α2-macroglobulin, α1-antitrypsin, C reactive peptide and complement factor C3. An increase in fibrinogen, an acute phase reactant, was also observed in tularemic European brown hares [33]. Other proteins such as albumin decrease. It is clear that *F. tularensis*-inoculated hares in this experiment responded to the tularemic sepsis via the above-described changes in protein metabolism.

**Figure 3:**
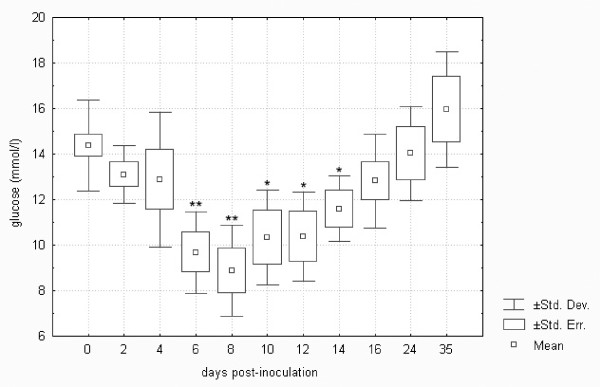
**Glucose in European brown hares on individual days post-inoculation with *F. tularensis***. See Figure 1 for a detailed description of groups.

As shown in Figure 3, glucose in *F. tularensis*-inoculated hares decreased to about 60% of the normal level on day 8 post-infection. The control glucose levels were consistent with published data [34]. A similar response was found in BALB/c mice and common voles infected with tularemia. Glucose levels in these two species of rodents significantly decreased from day 1 post-infection, which is characteristic of severe sepsis as well as hepatocellular damage [18].

**Figure 4:**
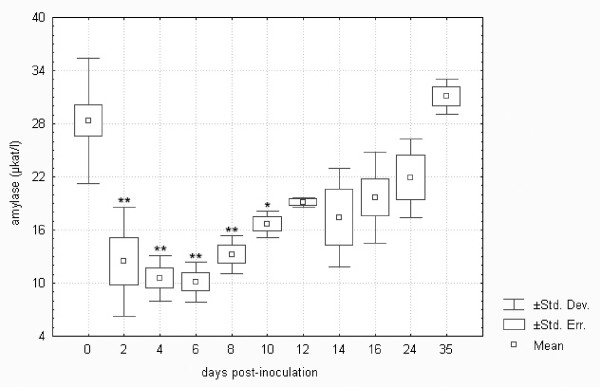
**Amylase in European brown hares on individual days post-inoculation with *F. tularensis***. See Figure 1 for a detailed description of groups.

Figure 4 demonstrates the significant decrease in amylase in *F. tularensis*-inoculated hares, declining to nearly 35% of the normal level. This enzyme catalyses the hydrolysis of polysaccharides and is associated with glycemia [35]. The decrease in both glucose and amylase demonstrates impairment of the energetic metabolism as tularemic sepsis develops.

**Figure 5:**
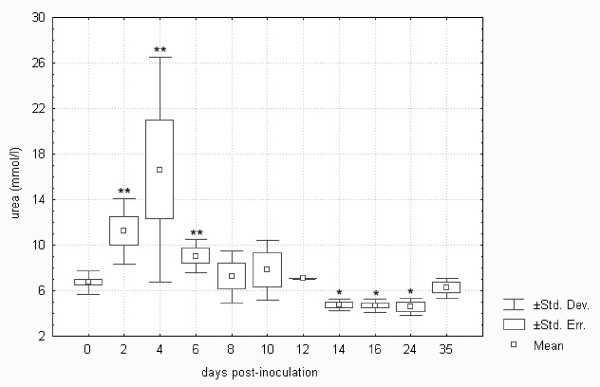
**Urea in European brown hares on individual days post-inoculation with *F. tularensis***. See Figure 1 for a detailed description of groups.

There was an increase in urea on days 2 to 6 post-infection (cf. Figure 5). This may be attributed to the fever and increased catabolism, as reported previously [18,32]. As shown, however, there was also a decrease in urea on days 14 to 24 post-infection that may have been due to hepatic insufficiency. Impaired hepatic function was also responsible for the nearly two-fold increase in total bilirubin on day 6 post-infection (p < 0.05).

**Figure 6:**
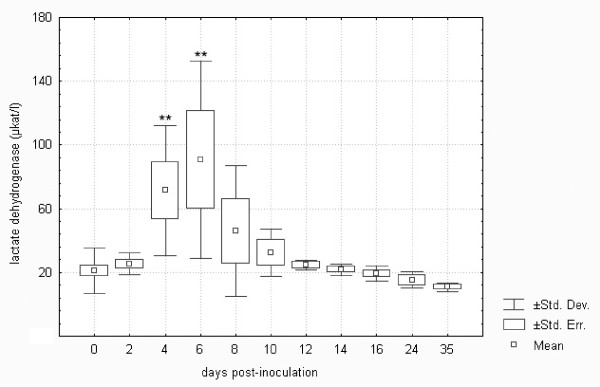
**Lactate dehydrogenase in European brown hares on individual days post-inoculation with *F. tularensis***. See Figure 1 for a detailed description of groups.

**Figure 7:**
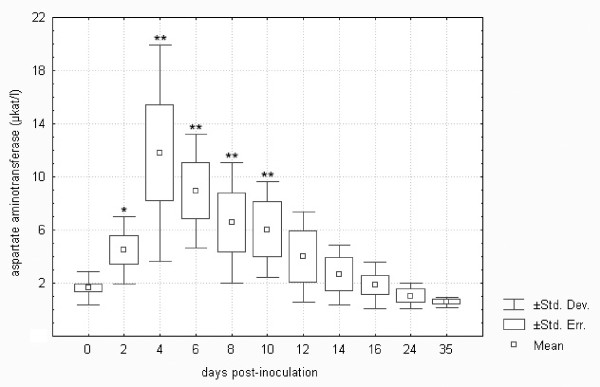
**Aspartate aminotransferase in European brown hares on individual days post-inoculation with *F. tularensis***. See Figure 1 for a detailed description of groups.

**Figure 8:**
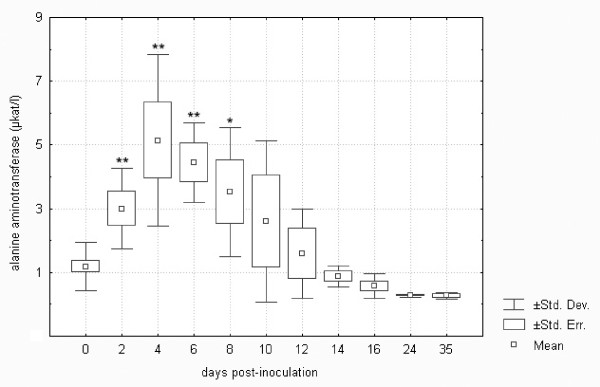
**Alanine aminotransferase in European brown hares on individual days post-inoculation with *F. tularensis***. See Figure 1 for a detailed description of groups.

Tularemia in *F. tularensis*-inoculated hares induced an almost four-fold elevation of lactate dehydrogenase of statistical significance on days 4 and 6 (cf. Figure 6). It is known that lactate dehydrogenase may be used to follow the progress of liver disease because it changes quickly. In an experimental study on the responses of BALB/c mice and common voles to tularaemia, lactate dehydrogenase started to rise earlier than aspartate aminotransferase and alanine aminotransferase and was considered an important indicator of acute hepatocellular damage in tularemia [18]. In the European brown hare, however, statistically significant increases in both aspartate aminotransferase and alanine aminotransferase were demonstrated at an earlier stage, i.e., from day 2 post-infection (cf. Figures 7 and 8). The elevation of aspartate aminotransferase levels in tularemic hares was more pronounced, increasing as much as seven-fold when compared with controls. Alkaline phosphatase remained unchanged, as in *F. tularensis *infection in rodents [18]. The above pattern of early hepatic lesions in tularemia has previously been demonstrated. Hepatic dysfunction in tularemia is probably a contributor to the morbidity and mortality of this infection [14] because the liver is considered to be of major importance in the body's defence mechanism against bacteria [36]. Although some authors observed a lack of positive correlations between the degree of hepatic damage and liver function tests [20], others demonstrated significant correlations between tissue bacterial burdens and biochemical parameters such as lactate dehydrogenase, alanine aminotransferase and glucose [18]. It is clear from Figures 6 to 8 that the trends for the three above-mentioned liver enzymes in the *F. tularensis*-inoculated group of hares were very similar.

Kidney function in *F. tularensis*-inoculated hares was within the normal limits because creatinine was only insignificantly elevated on days 2 and 4 and the changes in urea (cf. Figure 5) were due to liver impairment rather than kidney failure.

**Figure 9:**
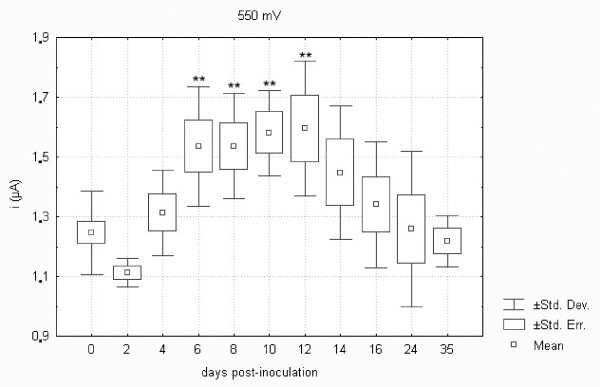
**Low molecular weight antioxidants oxidizable at a potential of 550 mV (i.e. uric and ascorbic acids) in European brown hares on individual days post-inoculation with *F. tularensis***. See Figure 1 for a detailed description of groups.

**Figure 10:**
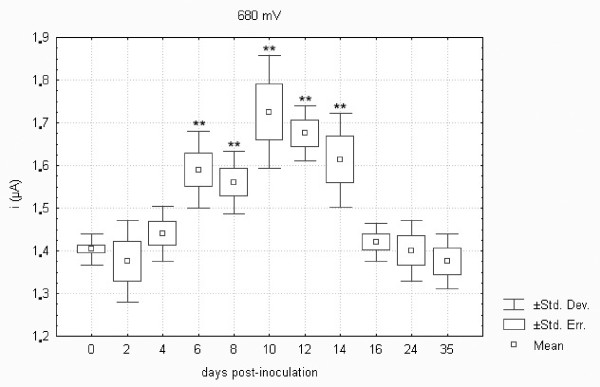
**Low molecular weight antioxidants oxidizable at a potential of 680 mV (i.e. glutathione) in plasma samples of European brown hares on individual days post-inoculation with *F. tularensis***. See Figure 1 for a detailed description of groups.

Low molecular weight antioxidants (LMWAs) in plasma samples collected from control and *F. tularensis*-inoculated hares were assayed using a screen-printed electrochemical sensor and square wave voltammetry (SWV). The LMWAs present in the sample appear as a typical wave in the anodic range when assayed by voltammetry [16]. Two peaks were found when assaying plasma samples collected from hares. The lower was found at 0.55 V, and the higher at 0.68 V. As previously described elsewhere, the first peak corresponds with uric and ascorbic acids [16], while glutathione is responsible for the second peak [37]. Figures 9 and 10 demonstrate the differences in the development profile of uric and ascorbic acids and glutathione, respectively. After an initial drop in both uric and ascorbic acids and glutathione on day 2 there was a statistically significant increase on days 6 to 12 and 6 to 14 post-inoculation. As shown, the LMWAs increased to about 120% of the normal level. These parameters were found to normalize from day 16 post-infection. A total of three LMWAs were estimated using SWV. However, the total number of chemical antioxidants occurring in the body is much higher [38]. The limit of detection of isolated compounds is in the range of 1-10 μM. This range of sensitivity is sufficient for determining the physiological concentrations of biologically relevant scavengers. It may be hypothesized that the increase in glutathione levels as a response to oxidative stress conferred by the *F. tularensis *infection further promotes its multiplication because this antioxidant provides the essential source of cysteine required for the growth and proliferation of *Francisella *[17].

Reactive nitrogen species (RNS) and reactive oxygen species (ROS) are intermediates that are involved in the host defence against various intracellular pathogens including *F. tularensis*. The production of reactive molecular species is induced in macrophages when they are exposed to pro-inflammatory cytokines, including IFN-γ and TNF-α. After activation, macrophages are capable of arresting bacterial replication [39]. *F*. *tularensis *is exposed to ROS and RNS not only in macrophages but also in other cell types or extracellularly in vivo, and both *F. tularensis tularensis *and *holarctica *subspecies are assumed to be virulent as they are armed with a variety of enzymes that can combat host ROS- and RNS-mediated killing mechanisms [40]. These processes may result in the peroxidation of cellular lipids due to hydroxyl radical production. It is possible to evaluate lipid peroxidation as a measure of oxidative damage.

**Figure 11:**
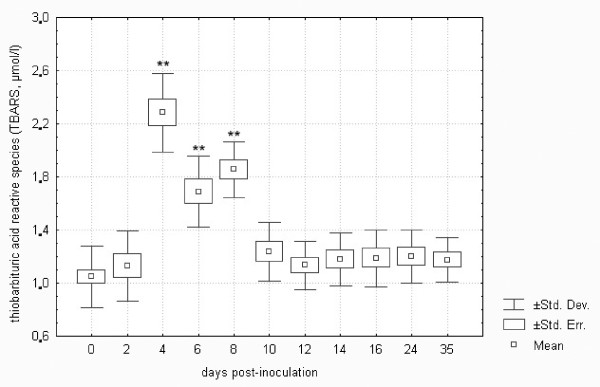
**Lipid peroxidation assessed as total thiobarbituric acid reactive species (TBARS) in plasma samples of European brown hares on individual days post-inoculation with *F. tularensis***. See Figure 1 for a detailed description of groups.

As shown in Figure 11, there was about a two-fold increase in lipid peroxidation assessed as total thiobarbituric acid reactive species (TBARS) in the *F. tularensis*-inoculated group of European brown hares on days 4 to 8 post-inoculation. From day 10, the TBARS level returned to within the normal range, probably due to the protective action of increased antioxidants (cf. Figures 9 and 10). The normal levels of TBARS in healthy European brown hares have not yet been reported. The TBARS of control hares in the present study ranged from 0.81 to 1.54 μmol/l. These values are similar to those found in humans (1.20 ± 0.30 μmol/l) [41].

Statistical analysis revealed a significant correlation between the uric acid levels measured using standard procedures of dry chemistry and LMWAs oxidizable at a potential of 550 mV (represented by the content of uric and ascorbic acids) assayed using square wave voltammetry in European brown hares on individual days post-inoculation with *F. tularensis *as well as in controls from days 0 to 35 (*n *= 96, *R *= 0.57, *p *= 0.01). LMWAs such as uric and ascorbic acids and glutathione were assayed using square wave voltammetry and the results obtained were consistent with the development of parameters measured by standard biochemical procedures. The correlation between TBARS and LMWAs oxidizable at a potential of 550 mV (i.e. uric and ascorbic acids) was statistically significant (*n *= 96, *R *= 0.30, *p *= 0.02). Thus the above results validated data obtained by square wave voltammetry in the present study.

Comparison of biochemistry profiles from hares surviving or succumbing to the *F. tularensis *infection is another important issue. Figures 1 to 11, however, demonstrate that there was a general trend in the development of each biochemistry parameter after the *F. tularensis *inoculation, common to survivors and dying hares.

The biochemical responses of various hosts to the development of tularemia were recently reported to vary. Differences in lipid metabolism (triglycerides and total cholesterol) were found in highly susceptible BALB/c mice and common voles [18] but, interestingly, not in the European brown hare in the present study. It seems that the European brown hare may be considered to have relatively lower susceptibility to tularemia. It is necessary to distinguish serologically positive specimens with and without clinical signs of the disease. Antibodies against *F. tularensis *may be recognized as early as 7 days post-infection [25]. Mortality in the present study occurred no later than 9 days post-infection in the European brown hare, and hares surviving to day 35 post-inoculation were free of *F. tularensis *based on culture and the mouse inoculation test. In our opinion, conditions leading to the development of clinical tularemia in hares should be further studied. Apart from vector-borne transmission, there are other routes of infection such as drinking contaminated water or the inhalation of infectious aerosols. It may be hypothesized that co-exposure of hares to other stressors may enhance their susceptibility to infection. The role of the tick salivary gland extract in accelerating and enabling the proliferation of *F. tularensis *in the host has also been recognized [42].

## Conclusions

Low molecular weight antioxidants such as uric and ascorbic acids and glutathione were assayed using square wave voltammetry and the results obtained were consistent with the development of parameters measured by standard biochemical procedures. Contrary to all expectations, the results of the present study show that the European brown hare may be considered as having relatively low susceptibility to tularemia. Therefore, the circumstances of tularemia in hares under natural conditions should be studied further.

## Competing interests

The authors declare that they have no competing interests.

## Authors' contributions

HB carried out the whole experiment, performed data analyses and took part in preparing the manuscript. JP planned the experiment, performed statistical analyses and participated in preparing the manuscript. VD, LP and JS prepared experimental animals, assisted in planning the experimental design and biochemical evaluations. MP and KV analysed plasma samples using cyclic voltammetry. FT and FV cultured *Francisella tularensis*, prepared the strain for experimental infection and quantified the bacterium in tissues. They also took part in preparing the manuscript. All authors contributed to the study design, the preparation of the manuscript and also read and approved the final manuscript.
